# Supplementary material for: Investigating the dynamics of climate finance disbursements: A panel data approach from 2003 to 2022
Source: PLoS One. 2025 Mar 7;20(3):e0318170. doi: 10.1371/journal.pone.0318170 (PMC11888150; doi:10.1371/journal.pone.0318170)
Supplement: Attachment — Submitted filename: Respsonse on first (2) Reviewers Comments.pdf [file pone.0318170.s003.pdf]

FEEDBACK ON REVIEWERS' REMARKS REGARDING THE ARTICLE

TITLE: Investigating the Dynamics of Climate Finance Disbursements: A Panel Data Approach from 2003 to 2022

No.	Concern of the reviewer	Response of the author (Action taken)	Evidence of the action taken
1	Create specific section for METHOD and organize into sub-sections as follows (1) Type and Source of Data (example, this study employs largely secondary data....) (2) Research Method and Model Specifications etc.	Thank you for your constructive feedback and suggestions for organizing the methodology section of our manuscript. In response to your comment, we have restructured the methodology section as per your recommendations to enhance clarity and comprehensiveness.<br><br>We have renamed the section to **"Research Methodology"** and organized it into the following sub-sections:<br>1. **Type and Source of Data**: This sub-section provides a detailed description of the nature of the data used in the study, emphasizing that the study primarily employs secondary data, along with details about the sources and relevant data collection processes.<br>2. **Model Specifications**: This sub-section outlines the theoretical framework and model specifications used in the analysis, explaining the dependent and independent variables and their expected relationships.<br>3. **Estimation Procedure**: This sub-section describes the statistical techniques and procedures employed to estimate the models, including diagnostic tests and robustness checks to ensure the validity of the results.<br><br>We believe that this revised structure better aligns with standard academic practices and improves the overall	See line-numbers: 397-436

		organization of the manuscript. We appreciate your valuable input, which has significantly contributed to improving the quality and readability of our paper.	
2	Change Discussion section to RESULTS AND ANALYSIS (DISCUSSION) - Must tally with the aims/purposes of the study - Please explain more on the issue of inclusivity (environmental justice framework) which involved community access and participation in climate change initiatives.	Thank you for your insightful comments and recommendations aimed at improving our manuscript. In response to your suggestions, we have made the following revisions:<br>• **Renamed the Discussion Section**: We have changed the "Discussion" section to **"Results and Analysis (Discussion)"** to ensure it aligns closely with the aims and purposes of our study. This restructuring provides a clearer connection between our findings and the research objectives outlined earlier in the manuscript.<br>• **Expanded on Inclusivity and Environmental Justice**: We have enhanced our discussion on the issue of inclusivity within the environmental justice framework. Specifically, we have included more detailed explanations on how community access and participation are integral to climate change initiatives. This addition emphasizes the importance of involving local communities in environmental decision-making processes and highlights how inclusivity can lead to more effective and equitable climate action.<br>These revisions aim to address your concerns and improve the overall clarity and depth of our analysis. We believe that the enhanced discussion provides a more comprehensive understanding of the subject matter and better reflects the goals of our research.<br>Please refer to the revised manuscript to see these changes in detail. We are grateful for your valuable feedback,	See line-numbers: 776-778

		which has significantly contributed to strengthening our work.<br>Thank you once again for your time and consideration.	
3	So many IN-TEXT CITATIONS but not addressed in the References section	Thank you for bringing to our attention the issue regarding in-text citations not being addressed in the References section. We have carefully reviewed and cross-checked all the in-text citations in the manuscript against the References section.<br>In the revised manuscript, we have ensured that:<br>• Every in-text citation is accurately listed in the References section.<br>• Any missing references have been added, and any redundant or irrelevant references have been removed.<br>• The formatting of both in-text citations and the References section adheres to the required citation style.<br>We appreciate your diligence in identifying this issue, and we believe these corrections enhance the credibility and completeness of our manuscript. Please feel free to review the updated version to verify these changes.<br>Thank you for your constructive feedback and support.	Check the revised Reference section
4	Most of the FIGURES and TABLE do not have their sources cited.	Thank you for your observation regarding the missing source citations for figures and tables in the manuscript.<br><br>We would like to clarify that all the tables and figures presented in the manuscript are built directly from the dataset used for our empirical analysis.<br><br>To ensure transparency and consistency, we have updated the manuscript to explicitly indicate this in the captions of all tables and figures. For example, the captions now	See line-numbers: 493-495

		include the statement: **"Source: Author's Analysis based on the dataset used for this study."**<br><br>We appreciate your attention to this detail, as it has allowed us to provide additional clarity and improve the quality of our work.	
5	Some REFERENCES are not formatted properly and have missing details (year, pages, publisher, DOI)	Thank you for highlighting the issue of improperly formatted references and missing details in the manuscript. We have thoroughly reviewed the References section and addressed these concerns as follows:<br>1. **Complete Information Added**: We have ensured that all references now include the necessary details, such as publication year, page numbers, publisher information, and DOI where applicable.<br>2. **Consistent Formatting**: The references have been reformatted to align with the required citation style, ensuring consistency throughout the section.<br>3. **Cross-Checked References**: We have cross-verified the details of all cited works to ensure accuracy and completeness.<br>These corrections enhance the professionalism and reliability of our manuscript. We appreciate your careful review and constructive feedback, which have been instrumental in improving the quality of our submission. Thank you once again for your valuable input.	Check the revised Reference section
6	The CONCLUSION of the study is somewhat disorganized, especially regarding the implications. It would be more effective to structure it into distinct sub-sections, such as Practical/Governance	Thank you for your insightful comments and recommendations aimed at improving our manuscript. In response to your suggestions, we have re-structured the conclusion section to reflect your recommendations, ensuring that it is clearer and more comprehensive. The revised conclusion section now includes the following components:	See line-numbers: 911-981

	Implications, Policy Implications, and Theoretical Implications. Please explain how could the issue of inclusivity i.e community participation and equitable access to climate change initiatives can be addressed by governments at all levels. Several key concepts can also be included in this issues notably multi-stakeholder engagement, empowering local governance, and implementing inclusive policy designs that prioritize vulnerable populations. Please provide some best practices and suggestions for improvement in this study (as stated earlier as the aims of this study). Based on the limitations of the study, please add suggestion for Future Studies.	1. **Objective of the Study**: A concise summary of the study's aims, emphasizing its focus on inclusivity and equitable access in climate change initiatives.<br>2. **Key Findings of the Study**: Highlights of our major discoveries, underscoring the importance of multi-stakeholder engagement, local governance empowerment, and inclusive policy designs.<br>3. **Practical and Governance Reflections**: Discussion on actionable strategies for governments at all levels to promote community participation and equitable access to climate initiatives. We particularly emphasize the role of empowering local governance structures, fostering partnerships with non-state actors, and ensuring resource distribution aligns with the needs of vulnerable populations.<br>4. **Policy Implications**: Recommendations for developing and implementing policies that embed inclusivity at their core, guided by principles of fairness, transparency, and equity.<br>5. **Theoretical Insights**: A synthesis of how the study contributes to the broader theoretical discourse on governance, community inclusion, and climate justice.<br>6. **Areas for Improvement and Future Directions**: Reflections on the study's limitations, such as limited case studies or regional scope, and suggestions for future research. For example, future studies could explore longitudinal impacts of inclusive policies or assess community-level outcomes across diverse geographic settings.<br>7. **Moving Forward**: A forward-looking discussion on how the findings can inform ongoing and future	

		climate action initiatives, encouraging sustained inclusivity and participation.<br>Your comments on inclusivity, community participation, and equitable access were particularly valuable in shaping the practical and governance reflections as well as the policy implications. We have addressed these issues by incorporating discussions on best practices such as multi-stakeholder engagement, empowering local governance through capacity-building initiatives, and designing policies that prioritize vulnerable populations through participatory mechanisms. For example, we suggest frameworks that ensure marginalized voices are not only heard but actively influence decision-making processes.<br>In addition, we have enriched the manuscript with suggestions for best practices, including:<br>• **Building Local Capacity**: Training programs for local governance to enable effective community-based climate solutions.<br>• **Transparent Resource Allocation**: Mechanisms for equitable resource distribution to address disparities.<br>• **Multi-Stakeholder Collaboration**: Encouraging partnerships across public, private, and non-governmental sectors to drive inclusive initiatives.<br>We appreciate your review and constructive feedback, which have been instrumental in enhancing the clarity, professionalism, and reliability of our manuscript. Thank you once again for your valuable input.	
7	The major reference from which the DATASET was obtained, notably the Climate Funds Update (CFU), is not properly cited in the References section	Thank you for your positive feedback and suggestions regarding the proper citation of the data source used for our empirical investigation. In response to your comments, we have added the following as a footnote, which is now reflected in the revised manuscript:	n.a

	at least please provide the website link/DOI link (if it's retrieved from jounals or official documents)	*Climate Funds Update, a respectable Washington-based think tank that tracks and analyzes climate finance. The data was obtained by means of their publicly accessible website, namely* [*https://climatefundsupdate.org*](https://climatefundsupdate.org). We appreciate your valuable input, which has meaningfully contributed to improving the quality and readability of our paper. Your thoughtful observations have helped us ensure proper acknowledgment of key resources, enhancing the transparency and credibility of our study. Many thanks.	
8	It is stated in the ABSTRACT "The FORECASTING results indicate a positive trend in disbursements from 2023 to 2027, with potential fluctuations driven by external factors, but missing reporting in the content.	Thank you very much for drawing our attention to the missing FORECASTING report mentioned in the abstract. We have addressed this oversight by adding the missing report in the empirical findings section of the revised manuscript. This section now includes a comprehensive analysis of the forecasting results, highlighting the positive trend in disbursements from 2023 to 2027 and discussing potential fluctuations influenced by external factors. We hope the revised manuscript reflects your suggestions and provides a clearer and more complete presentation of the findings. We are indebted to your critical reviewing, which has significantly enhanced the quality and coherence of our work. Many thanks.	n.a
9	PLOS authors have the option to publish the peer review history of their article (what does this mean?). If published, this will include your full peer review and any attached files. If you choose "no", your identity	We have no problem with publishing the peer review history of our article. Thank you very much.	n.a

	will remain anonymous but your review may still be made public.		
